# Paeoniflorin attenuates monocrotaline-induced pulmonary arterial hypertension in rats by suppressing TAK1-MAPK/NF-κB pathways

**DOI:** 10.7150/ijms.69289

**Published:** 2022-03-28

**Authors:** Min Yu, Xuecheng Wu, Jingjing Wang, Mengyu He, Honghao Han, Song Hu, Jian Xu, Mingxia Yang, Qi Tan, Yanli Wang, Hong Wang, Weiping Xie, Hui Kong

**Affiliations:** 1 Department of Respiratory and Critical Care Medicine, The Affiliated Suzhou Hospital of Nanjing Medical University, Suzhou Municipal Hospital, Gusu School, Nanjing Medical University, Suzhou, Jiangsu 215000, P.R. China.; 2 Department of Respiratory and Critical Care Medicine, the First Affiliated Hospital of Nanjing Medical University, Nanjing, Jiangsu 210029, P.R. China.; 3 Department of Respiratory Medicine, Shanghai Pulmonary Hospital, Tongji University School of Medicine, Shanghai 200433, P.R. China.; 4 Department of Respiratory Medicine, the Third Affiliated Hospital of Soochow University, Changzhou, Jiangsu 213003, P.R. China.; 5 Department of Respiratory and Critical Care Medicine, The Affiliated Changzhou No.2 People's Hospital of Nanjing Medical University, Changzhou 213003, P.R. China.

**Keywords:** paeoniflorin, pulmonary arterial hypertension, pulmonary vascular remodeling, endothelial-to-mesenchymal transition, smooth muscle cells, monocrotaline

## Abstract

Pulmonary arterial hypertension (PAH) characterized by pulmonary vascular remodeling is a lethal disease. Paeoniflorin (PF) is a monoterpene glycoside with numerous beneficial functions, such as vasodilation, anti-inflammation and immunomodulation. This study aims to investigate the effects of PF on monocrotaline (MCT)-induced PAH rats. Our data showed that both prophylactic or therapeutic administration of PF alleviated MCT-induced increasing of right ventricular systolic pressure (RVSP), prevented right ventricle hypertrophy and pulmonary arterial remodeling, as well as inhibited inflammatory cell infiltration around pulmonary arteries. Meanwhile, PF blocked MCT-induced endothelial-mesenchymal transition (EndMT) as indicated by the restored expression of endothelial markers in lung. Moreover, PF inhibited MCT-induced down-regulation of bone morphogenetic protein receptor 2 (BMPR2) and suppressed MCT-induced phosphorylation of transforming growth factor-β (TGFβ) activated kinase 1 (TAK1) in *vivo*. *In vitro* studies indicated that PF prevented human pulmonary arterial smooth muscle cells (PASMCs) from platelet-derived growth factor-BB (PDGF-BB)-stimulated proliferation and migration. PF also partially reversed TGFβ1, interleukin-1β (IL-1β) and tumor necrosis factor (TNF-α) co-stimulated endothelial-to-mesenchymal transition (EndMT) in cultured human pulmonary artery endothelial cells (HPAECs). Signaling pathway analysis demonstrated that the underlying mechanism might be associated with the inhibition of TAK1-MAPK/NF-κB pathways. Taken together, our results suggested that PF could be a potential drug for the treatment of PAH.

## Introduction

Pulmonary arterial hypertension (PAH) is a lethal disease characterized by sustained elevation of pulmonary arterial pressures, which results in right ventricular hypertrophy and ultimately right heart failure 1. The primary pathological feature of PAH is pulmonary vascular remodeling, comprising the injury and dysfunction of endothelial cells in intima, over-proliferation of smooth muscle cells in media, as well as collagen deposition and inflammatory cell infiltration in adventitia 2. In the past decade, remarkable progresses have been achieved in understanding the molecular mechanisms of PAH. Based on these progresses, newly developed targeted therapies acting on endothelin-1 receptor, prostacyclin, and nitric oxide pathways significantly improve the clinical outcome. Nevertheless, the long-term prognosis of PAH remains poor 3. Therefore, further therapeutic approaches are urgently required for better treatment of PAH.

Paeoniflorin (PF), a monoterpene glucoside, is the most abundant bioactive component of total glucosides of paeony extracted from the root of Paeonia lactiflora. In clinical practice, PF is widely used in the treatment of autoimmune diseases, such as rheumatoid arthritis and systemic lupus erythematosus. Increasing evidence reveals that PF have many beneficial pharmacological effects including anti-inflammation, immunoregulation, anti-oxidative, anti-tumor, neuroprotection, and analgesia 4, 5. Recently, it was reported that PF inhibited hypoxia-induced pulmonary artery smooth muscle cells (PAMSCs) proliferation via upregulating A_2B_ adenosine receptor *in vitro* 6. In various endothelial cells, PF was reported to protect against lipopolysaccharide, hydrogen peroxide, or radiation-induced injury 7, 8. Moreover, PF ameliorated BMPR2 down-regulation-mediated EndMT and thereafter prevented the development of chronic hypoxia/SU5416-induced PAH in rats 9. In bleomycin-induced pulmonary fibrosis in mice, it was reported that PF effectively suppressed extracellular matrix deposition in lung by inhibiting the activation of TGFβ/SMAD signaling. Together, these findings suggest that PF could be a potential drug for inhibiting vascular remodeling. However, the therapeutic effects and molecular mechanisms of PF should be further validated and investigated in other PAH animal models. Therefore, in the present study, the preventive and curative effects of paeoniflorin and the potential underlying mechanisms were investigated in a rat model of MCT-induced PAH.

## Materials and Methods

### Experimental animals

All experimental procedures were approved by the Institutional Animal Care and Use Committee of Nanjing Medical University (NJMU/IACUC-1809008) and were in accordance with the guidelines published by the National Institutes of Health Guide for the Care and Use of Laboratory Animals (publication No. 85-23, revised 1996). Male adult Sprague-Dawley rats (200-220 g) provided by Nanjing Medical University Experimental Animal Center (Nanjing, China) were housed under conditions of constant temperature and controlled illumination. Food and water were available *ad libitum*. After 1-week accommodation to the vivarium, rats were randomly assigned into three groups (Control, MCT, MCT+PF) to receive a single intraperitoneal injection of 1 mL 0.9% saline or 60 mg/kg MCT (Sigma-Aldrich, St. Louis, MO) on the first day. For the preventive study, PF (Nantong Jingwei biological science and Technology Co, Ltd, Nantong, China) was intragastric administration daily at the dosages of 100, 200, 300 mg/kg for 21 days from the first day. For the therapeutic study, PF (300 mg/kg) was intragastric administration at day 21 after MCT injection for 14 days.

### Hemodynamic measurements and specimen collection

Rats were anesthetized with an intraperitoneal injection of sodium pentobarbital (50 mg/kg) and hemodynamic parameters were measured by right-heart catheterization. The right ventricular systolic pressure (RVSP) and RV contractility (max dP/dT) was recorded using a PowerLab data acquisition system (ADI Instruments). After hemodynamic measurements, the lung tissues were excised for protein isolation and histological assessment. The right ventricular hypertrophy index was presented as the weight ratio of right ventricular to the left ventricle and interventricular septum (RV/LV+S).

### Morphological measurements

Lung tissue underwent saline perfusion through the pulmonary artery. The left lungs and right ventricular tissues were fixed in 4% paraformaldehyde, embedded in paraffin, and sectioned. After hematoxylin and eosin (H&E) staining or Masson trichrome staining was performed, sections were examined by a microscopic digital camera. Morphometric analysis was performed in the pulmonary arteries (external diameter: 30 - 150 μm) at a magnification of 400 ×. Pulmonary artery medial wall thickness (PAWT) was calculated as: medial wall thickness (%) = (external diameter - internal diameter)/external diameter × 100. For quantitative analysis, 20 randomly selected vessels from each rat were analyzed, and the average was calculated. The lung tissue sections were then stained with terminal-deoxynucleotidyl transferase mediated dUTP nick end labeling (TUNEL) (Roche, Switzerland) to evaluate the cell apoptosis. Thirty pulmonary arteries in each rat were randomly selected for detection of the percentage of apoptotic PASMCs. Right ventricular hypertrophy was measured by the cardiomyocyte cross-sectional area (CSA) in HE-stained sections. Cardiomyocyte fibrosis was determined by Masson trichrome staining. The degree of fibrosis in the lung and heart tissue was calculated by measuring the percentage of the collagen-positive area using Image-Pro Plus software.

### Immunohistochemistry studies

To assess the proliferation of PASMCs in small pulmonary arteries, α-smooth muscle actin (α-SMA) and proliferating cell nuclear antigen (PCNA) immunohistochemistry staining was performed. To evaluate the effects of PF on inflammatory cell infiltration in MCT induced PAH, monocyte/macrophage marker CD68 and mast cell tryptase staining were conducted. Generally, immunohistochemistry studies were performed using standard procedures. The sections were incubated at 4 °C overnight with α-SMA, PCNA, CD68 and tryptase (Abcam, 1:100) antibodies, respectively. Sections were then incubated with secondary antibodies and the immunoperoxidase reaction was visualized using diaminobenzidine as a chromogen (Vector Labs, USA). The muscularization of distal vessels was quantified, and the percentages of non-muscularized, partially muscularized, and fully muscularized vessels were calculated by α-SMA immunohistochemistry. The infiltration of macrophages and mast cells was evaluated by CD68 and tryptase immunohistochemistry method.

### Culture of PASMCs

Primary PASMCs were obtained from ScienCell (CA, USA) and maintained in smooth muscle cell medium (SMCM, ScienCell) with 2% fetal bovine serum, 1% smooth muscle cell growth supplement, and 1% penicillin/streptomycin solution, and sub-cultured in petri dishes (Corning 430167, diameter 100 mm, Corning, Inc., Tewksbury, MA, USA) at 5% CO_2_ and 37 °C. The cells of four-to-eight passages were used in the experiments. The culture medium was changed every 3 days and the cells were sub-cultured until confluence. For each experiment, cells were starved for 24 h in SMCM without growth supplement and serum before the experiment to synchronize the cells into quiescence and then treated with growth factors PDGF-BB (R&D Systems, Minnea-polis, USA, 20 ng/mL). For detecting intercellular signaling transduction, cells were harvested 5 min or 30 min after PDGF-BB challenge.

### Proliferation and apoptosis assays

PASMCs were seeded into a 96-well plate (5000 cells/well) for 24 h and were starved for next 24 h. PDGF-BB was added for 24 h in the presence or absence of indicated concentrations of PF (Sigma-AldrichSt, Louis, MO, USA) in non-serum SMCM. Cell counting kit-8 (CCK-8) assay was used to examine the cell viability of human PASMCs at 450 nm using a microplate reader (Thermo Scientific, CA, USA). Moreover, the effects of PF on the proliferation of PASMCs were further confirmed by measuring the incorporation of 5-ethynyl-20-deoxyuridine (EdU) with the EdU Cell Proliferation Assay Kit (Ribobio, Guangzhou, China). Five fields were randomly selected from each dish by fluorescence microscopy (DM2500; Leica, Wetzlar, Germany).

Flow cytometry analysis was performed to assess the levels of cell apoptosis. In brief, the cells were lysed with 0.25% trypsin, and then collected by centrifugation. The cells were washed twice in PBS buffer and resuspended at 1× annexin-V binding buffer. Cells were then stained with Annexin V-FITC and propidium iodide (PI) at room temperature. After incubation in the dark for 30 min, the cells were subjected to flow cytometry and the rate of cell apoptosis was determined.

### Cell migration assays

Transwell chamber assay and wound healing assay were used to assess the PASMCs migration. First, we used a 24-well Transwell chamber (Corning, Corning, NY, USA) to examine the PDGF-BB-induced PASMCs migration. PASMCs (5 × 10^4^ cells) were starved for 24 h and were loaded into the upper chamber. In the lower chamber, PDGF-BB was added to non-serum SMCM with different concentrations of PF (0.1, 1, 10 μM). After 24 h, the upper surface of the membrane was scraped using a cotton swab and cells on the lower surface were fixed in methanol for 30 min and stained with 5% crystal violet for 30min. For each filter, at least 5 randomly chosen fields were imaged to obtain a total cell count.

For wound healing assay, PASMCs were plated in 6-well plates and grew to confluence. Then, the cell media was replaced by serum-free media for 24 h. A plastic pipette tip was drawn across the center of the plate to produce a clean wound area. Multiple photographs of the wound were obtained 24 h post-wounding. The migration distance between the leading edge of the migrating cells and the edge of the wound was measured for comparison.

### Culture of HPAECs

Human pulmonary artery endothelial cells (HPAECs), obtained from Lonza Group (Switzerland), were maintained in complete endothelial cell growth medium-2 (Lonza) at 5% CO_2_ and 37 °C. The cells of three-to-six passages were used in the experiments. The HPAECs were stimulated by recombinant human TGFβ1 (R&D Systems, 5 ng/ml), IL-1β (R&D Systems, 0.1 ng/ml) and TNF-α (R&D Systems, 5 ng/ml) with or without paeoniflorin (10 μM) treatment 10. For detecting intercellular signaling transduction, cells were harvested 30 min after TGFβ1, IL-1β and TNF-α challenge, while for other detections, cells were harvested 48 h after TGFβ1, IL-1β and TNF-α challenge.

### Immunofluorescence Staining

Immunofluorescence staining was performed as previously described 11. Sections or HPAECs were incubated with anti-CD31 antibody (Proteintech, USA), anti-vascular endothelial cadherin (VE-cadherin) antibody (Abcam, Cambridge, MA) and anti-α-SMA (Abcam) antibody overnight at 4°C, then secondary antibody for 2 h at 37°C. Nuclei were stained with 4, 6-diamidino-2-phenylindole (DAPI, Sigma-Aldrich). Fluorescence images were captured by use of the Leica TCSSP5 confocal system. All sections were examined and at least three to five images from each section were acquired.

### Western blotting analysis

Total proteins were extracted from the lung tissues, or cultured PASMCs and HPAECs with RIPA Lysis and Extraction buffer (Thermo Scientific, Rockford) containing protease and phosphatase inhibitor cocktail (Roche Company, Germany) for western blotting. Equal amounts of protein (50 μg) was subjected to electrophoresis on 10% SDS-PAGE gels and transferred to PVDF membranes (Millipore) and then probed with primary antibodies, followed by incubation with the corresponding secondary antibodies. Antibodies against eNOS, p-p38/p38 MAPK, p-ERK1/2/ERK1/2, p-TAK1/TAK1 and NF**-**κB p-p65/p65 were obtained from Cell Signaling Technology (Beverly, MA, USA). Antibodies against VE-cadherin, α-SMA, Vimentin and BMPR2 were obtained from Abcam. TGFβ and β-actin antibody was obtained from Proteintech. Protein signal was detected using the WesternBright ECL (Advansta, CA). The band intensity was quantified by Quantity One software (Bio-Rad, USA).

### RT-qPCR

RNA derived from lung tissues was extracted with Trizol reagent (Gibco BRL, Grand Island, NY, USA). Reverse transcription was performed with 1000 ng of total RNA with SYBR Premix Ex Taq^TM^ (TaKaRa, Japan). Quantitative Real-time PCR (RT-qPCR) was conducted using ABI Prism 7500 FAST apparatus (Applied Biosystems, Foster City, CA, USA) according to the manufacturer's instructions. The primer sequences are listed in *Table 1*. The 2^-ΔΔCt^ method was used to quantify mRNA expression relative to β-actin.

### Statistical analysis

Data were analyzed in SPSS 18.0 software (SPSS Inc., Chicago, IL, USA) by Student's t-tests or one-way ANOVA followed by Fisher's LSD test. All data were expressed as mean ± standard error of the mean (SEM). Statistical significance was defined as P < 0.05.

## Results

### Effects of preventive treatment of PF on RVSP and pulmonary vascular remodeling in the MCT-induced PAH

**Figure 1:**
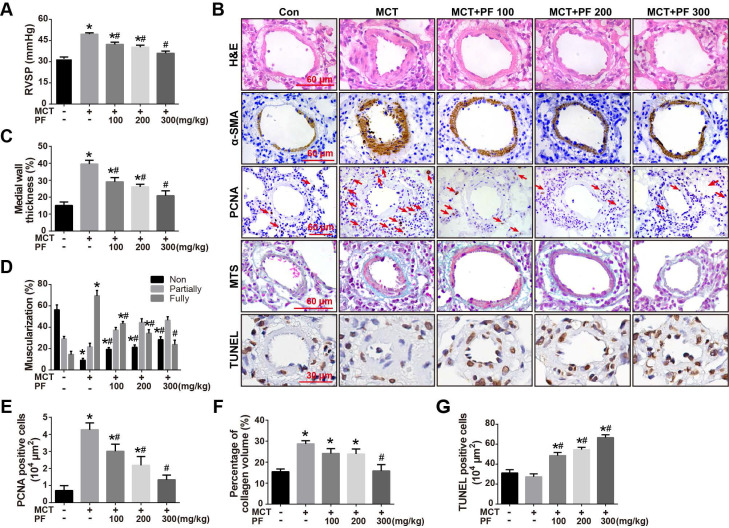
Effects of preventive treatment of PF on RVSP and pulmonary vascular remodeling in the MCT-induced PAH. (A) PF prevented MCT-induced increment of RVSP (n = 6-10). (B) Representative images of H&E staining, Masson's trichrome staining, immunohistochemistry for α-SMA, PCNA and TUNEL staining in lungs. (C) Quantifications of pulmonary artery medial wall thickness index in lungs. PF markedly reduced MCT-induced medial wall thickness of pulmonary artery (n = 6-10). (D) PF quantitatively attenuated MCT induced pulmonary vascular muscularization. Nonmuscularized vessels (Non), partially muscularized vessels (Partially), and fully muscularized vessels (Fully) were analyzed (n = 6-8). (E) Quantification of perivascular PCNA-positive cells (n = 6-8). (F) PF (300 mg/kg) inhibited MCT-induced adventitial collagen deposition (n = 6-8). (G) PF treatment markedly increased the percentage of apoptotic cells in MCT-induced PAH rats. (n = 6-8). Data were indicated as the mean ± standard error (SEM): (∗) P < 0.05 vs control; (#) P < 0.05 vs MCT.

To investigate the preventive efficacy of PF in experimental PAH, rats were treated with a single intraperitoneal injection of MCT (60 mg/kg) and PF was daily administrated on the same day and last for 21 days. Hemodynamic data showed that RVSP in rats of MCT-treated group was significantly higher than those of control group. Preventive treatment of PF suppressed MCT-induced elevation of RVSP in a dose-dependent manner (Figure 1A).

The effect of PF on pulmonary vascular remodeling was first examined by H&E staining. As shown in Figures 1B and 1C, the pulmonary artery medial wall thickness was significantly thickened in MCT-treated PAH rats, which was alleviated by PF. Small pulmonary artery (external diameter, 30 - 150 μm) muscularization was determined by α-SMA immunostaining. The number of fully muscularized arteries in MCT group was significant more than those in control group (Figures 1B and 1D), which was dose-dependently reversed by the treatment of PF. PCNA immunostaining showed that preventive treatment of PF inhibited MCT-induced cell proliferation around small pulmonary arteries (Figures 1B and 1E). Pulmonary vascular adventitial fibrosis was evaluated by Masson trichrome staining of collagen. Figures 1B and 1F showed that MCT treatment induced significant collagen deposition in the distal pulmonary arteries, which was reversed by the administration of PF at 300 mg/kg. Cell apoptosis around pulmonary arteries was detected using TUNEL staining in sections. As shown in Figure 1B and 1G, no significant difference was found in the number of apoptotic cells between the MCT group and control group. However, treatment of PF markedly increased the number of apoptotic cells around the pulmonary vascular in MCT-induced PAH rats. Together, these results demonstrate that PF could prevent the development of MCT-induced PAH and inhibited pulmonary vascular remodeling.

### Effects of preventive treatment of PF on right ventricular remodeling in the MCT-induced PAH

**Figure 2:**
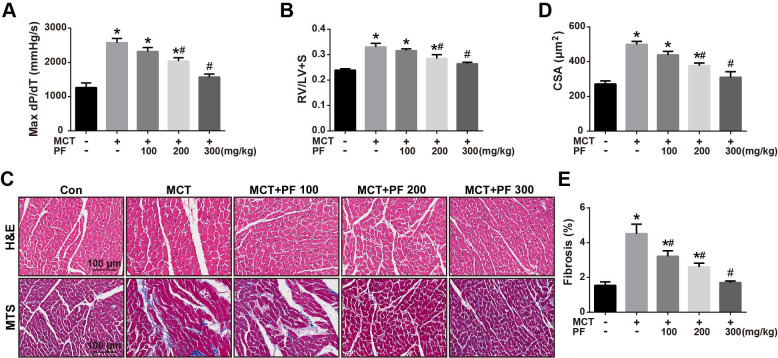
Effect of preventive administration of PF on MCT-induced right ventricular remodeling. (A) PF prevented MCT-induced elevation of max dP/dT (n = 6-8). (B) PF prevented MCT-induced increment of right ventricular hypertrophy (RV/LV+S) (n = 6-10). The effect of PF on cardiomyocyte hypertrophy and fibrosis was determined by H&E staining and Masson trichrome staining. (C) Representative images of H&E staining and Masson trichrome staining in RV sections. (D) PF prevented MCT-induced elevation of cardiomyocyte cross-sectional area (CSA) (n = 6-8). (E) PF attenuated right ventricle fibrosis in MCT-induced PAH rats (n = 6-8). Data were presented as mean ± SEM: (∗) P < 0.05 vs control; (#) P < 0.05 vs MCT.

We then investigate the efficacy of PF on right ventricular remodeling and function in experimental PAH *in vivo*. Hemodynamic data showed that RV contractility (max dP/dT) in rats of MCT-treated group was significantly higher than those of control group. Preventive treatment of PF suppressed MCT-induced elevation of max dP/dT (Figure 2A). Additionally, PF also inhibited MCT-induced right ventricular hypertrophy dose-dependently (Figure 2B). The effect of PF on cardiomyocyte hypertrophy and fibrosis was determined by H&E staining and Masson trichrome staining. As shown in Figures 2C and 2D, a significant elevation of CSA was observed in the right ventricle of MCT-PAH rats, which was markedly decreased by PF. Similarly, PF also attenuated right ventricle fibrosis in MCT-induced PAH rats (Figures 2C and 2E).

### Effect of preventive administration of PF on MCT-induced inflammation in lung

**Figure 3:**
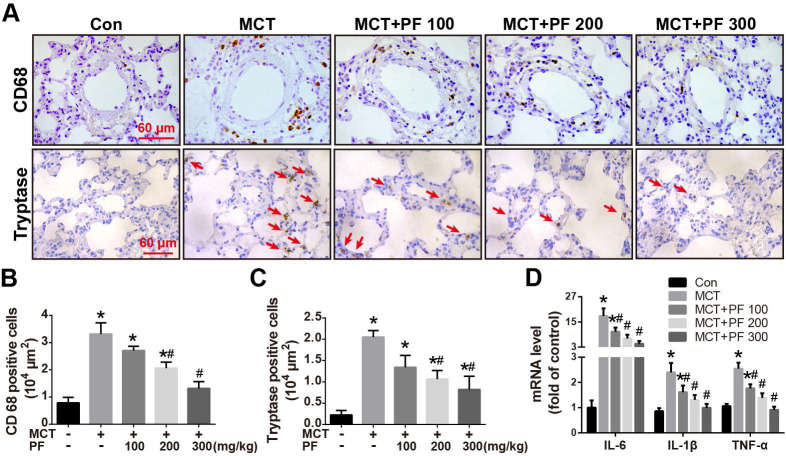
Effect of preventive administration of PF on MCT-induced inflammation in lung. (A) Representative immunofluorescent images of CD68 and tryptase in lungs. (B) Quantification of perivascular CD68-positive cells (n = 6-8). (C) Quantification of perivascular tryptase-positive cells (n = 5-8). (D) PF prevented MCT-induced high transcription of inflammatory mediator (IL-6, IL-1β, TNF-α) mRNA (n = 6-8). Data were presented as mean ± SEM: (∗) P < 0.05 vs control; (#) P < 0.05 vs MCT.

Immunostaining for macrophage/monocyte (CD68) and mast cell (tryptase) were performed to determine the inflammatory cell infiltration in lung. Compared to the control group, single MCT injection resulted in an intensive infiltration of macrophages/monocytes and mast cells around pulmonary arteries. This MCT-induced inflammatory cell infiltration was significantly alleviated by PF at 200 mg/kg or 300 mg/kg daily (Figures 3A-C). Furthermore, mRNA levels of inflammation related cytokines/chemokines were determined by RT-qPCR. As shown in Figure 3D, PF significantly inhibited MCT-induced transcription of IL-6, IL-1β and TNF-α dose-dependently.

### Effect of preventive administration of PF on MCT-Induced Endothelial-to-Mesenchymal Transition (EndMT) *in vivo*

**Figure 4:**
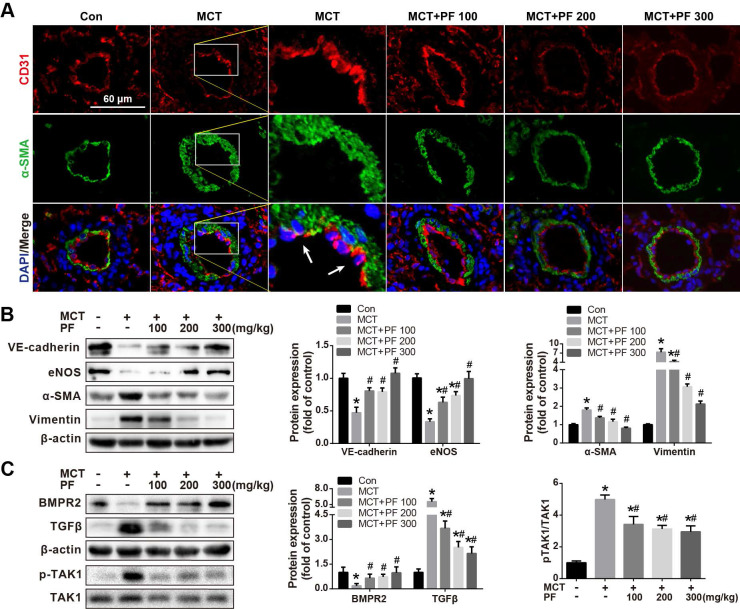
Effect of preventive administration of PF on MCT-induced endothelial-to-mesenchymal transition in lung. (A) Representative fluorescent microscopy images of CD31 and α-SMA costaining in the lungs (n=3). Green fluorescence represents α-SMA, blue fluorescence is 4,6-diamidino-2-phenylindole nuclei staining, and red fluorescence represents CD31. (B) Representative images and analysis of blotting for VE-cadherin, eNOS, α-SMA and Vimentin in lungs. PF markedly decreased MCT-induced overexpressions of α-SMA and Vimentin and partially reversed the expression of VE-cadherin (n=4) and eNOS (n=6) in lungs. (C) Representative images of blotting for BMPR2, TGFβ1 and phosphorylation of TAK1 in lungs. PF partially or completely reversed the expression of BMPR2 (n=6), TGFβ1 (n=4) and TAK1 phosphorylation (n=5). Data were presented as the mean ± SEM: (∗) P < 0.05 vs control; (#) P < 0.05 vs MCT.

Double immunofluorescence staining for CD31 and α-SMA in lung tissue sections was used to evaluate the effect of PF on MCT treatment induced EndMT *in vivo*. As shown in Figure 4A, CD31and α-SMA double positive staining (CD31^+^/α-SMA^+^) cells were found in the inner of pulmonary arteries from MCT-treated mice. However, CD31^+^/α-SMA^+^ cells were barely found in control group or PF-treated groups. These results suggested that PF could inhibit EndMT in MCT-induced rat model of PAH. To validate this result, protein levels of EndMT-related markers including VE-cadherin, eNOS, α-SMA and Vimentin in lung were determined by western blot. Our data indicated that preventive PF administration restored MCT-induced down-regulation of endothelial marker VE-cadherin and eNOS, while suppressed MCT-induced up-regulation of mesenchymal marker α-SMA and Vimentin (Figure 4B).

### Effect of preventive administration of PF on bone morphogenetic protein receptor type 2 (BMPR2) and TGFβ expression in lung in MCT-Induced PAH

The expression of BMPR2, a member of TGFβ-receptor superfamily, is reported to be down-regulated in various forms of PAH 12, 13. In the current study, single MCT injection resulted in a significant down-regulation of BMPR2 protein expression in lung, which was restored by the preventive administration of PF. Additionally, TGFβ, an important cytokine involved vascular remodeling, was up-regulated by MCT injection, which was significantly inhibited by the treatment of PF dose-dependently (Figure 4C). Considering that TGFβ-activated kinase 1 (TAK1) is a peculiar mitogen-activated protein kinase kinase kinase (MAPKKK) that mediates TGFβ and bone morphogenetic protein signaling, the phosphorylation and activation of TAK1 was further evaluated. As shown in Figure 4C, MCT induced a substantial phosphorylation of TAK1 in lung, which was significantly inhibited by preventive treatment of PF.

### Effects of therapeutic administration of PF on RVSP, pulmonary vascular remodeling and inflammatory response in MCT-induced PAH

**Figure 5:**
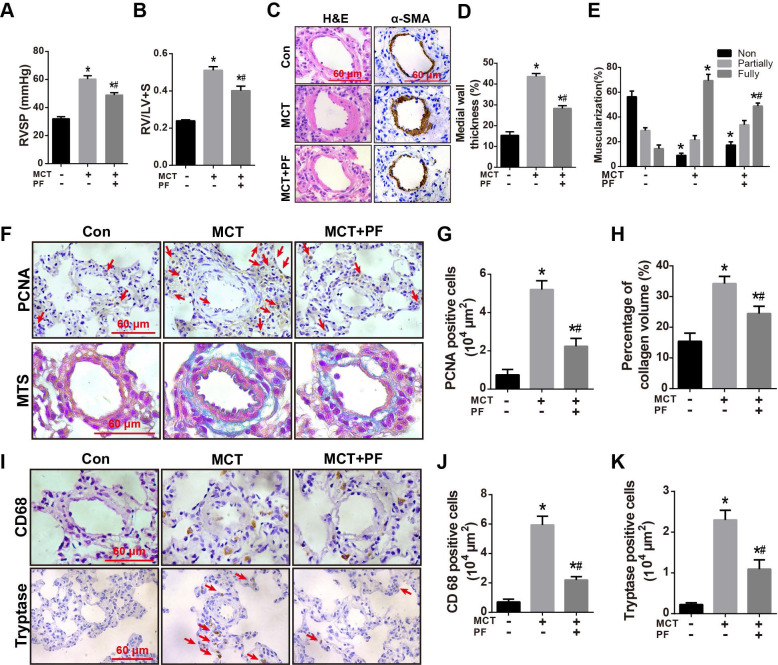
Effects of PF on RVSP, pulmonary vascular remodeling and inflammatory response in the therapeutic treatment of MCT-induced PAH. (A) and (B) PF partially reversed MCT-induced increment of RVSP and right ventricular hypertrophy (RV/LV+S) in the therapeutic experiments (n = 10-15). (C) Representative images of H&E staining and immunohistochemistry for α-SMA in lungs. (D) Quantifications of pulmonary artery medial wall thickness index in lungs. PF markedly reduced MCT-induced increase of medial wall thickness in muscular pulmonary arteries (n = 6-10). (E) PF quantitatively attenuated MCT induced the muscularization of small pulmonary arteries. Nonmuscularized vessels (Non), partially muscularized vessels (Partially), and fully muscularized vessels (Fully) were analyzed (n = 6-8). (F) Representative images of Masson's trichrome staining and immunohistochemistry for PCNA in lungs. (G) Quantification of perivascular PCNA-positive cells (n = 6-10). (H) PF (300 mg/kg) inhibited MCT-induced adventitial collagen deposition (n = 6-8). (I) Representative images of immunohistochemistry for CD68 and tryptase in lungs. (J) Quantification of perivascular CD68-positive cells (n = 6-8). (K) Quantification of perivascular tryptase-positive cells (n = 5-8). Data were indicated as the mean ± standard error (SEM): (∗) P < 0.05 vs control; (#) P < 0.05 vs MCT.

To investigate the therapeutic efficacy of PF in experimental PAH, rats were received a single intraperitoneal injection of MCT (60 mg/kg) while daily PF (300 mg/kg) administration was begun at day 21 and last for 14 days. As shown in Figures 5A and 5B, therapeutic administration of PF also significantly alleviated MCT-induced RVSP elevation and right ventricular hypertrophy (RV/LV+S).

H&E staining, α-SMA immunostaining, and PCNA staining indicated that therapeutic treatment of PF partially reversed MCT-induced thickening of PAWT (Figures 5C and 5D), muscularization of small pulmonary arteries (Figures 5C and 5E), and cell proliferation around pulmonary arteries (Figures 5F and 5G), respectively. Moreover, Masson trichrome staining showed that therapeutic administration of PF ameliorated MCT-induced collagen deposition in adventitia (Figures 5F and 5H).

Inflammatory response in MCT-induced PAH was evaluated by macrophage/ monocyte and mast cell infiltration around small pulmonary arteries. As shown in Figures 5I-K, CD68 and tryptase immunostaining demonstrated that therapeutic administration of PF inhibited MCT-induced infiltration of macrophages/ monocytes and mast cells around small pulmonary arteries.

### Effect of PF on PDGF-BB-induced PASMC proliferation, migration and apoptosis

**Figure 6:**
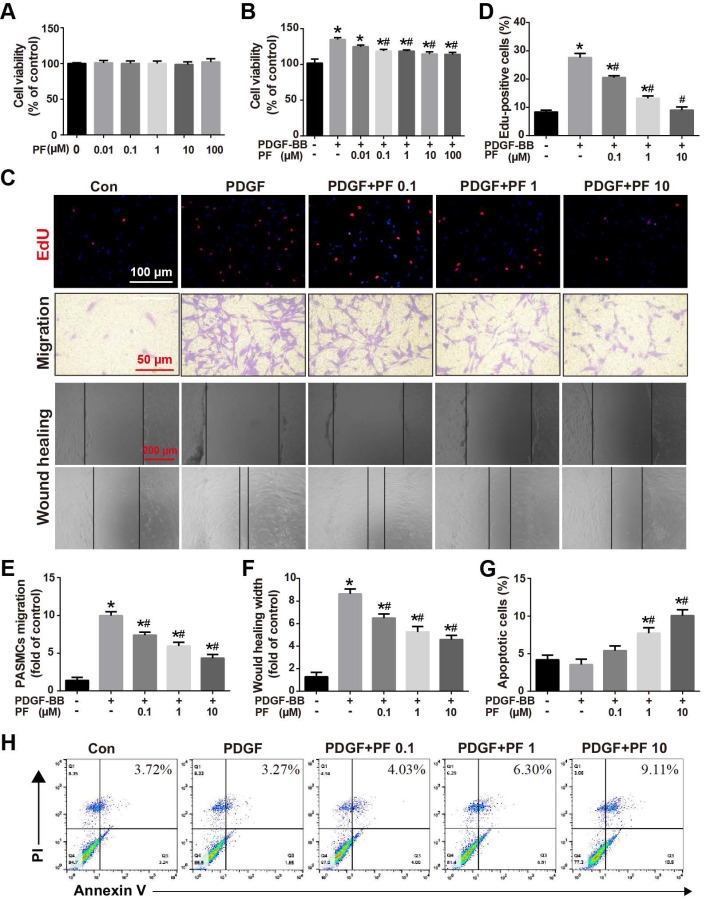
Effect of PF on PDGF-BB-induced human PASMCs proliferation and migration. (A) PF showed no cytotoxicity to PASMCs (n=4). (B) CCK-8 assay showed that PF inhibited PDGF-BB-induced cell proliferation in a dose-dependent manner (n=6). (C) Representative images of EdU (5-ethynyl-2ʹ-deoxyuridine) incorporation assay, transwell migration assay and scratch assay in PASMCs. (D) PDGF-BB-induced EdU-postive cells (red) were decreased by PF (n=3). (E) and (F) Transwell migration chamber assay and scratch assay showed that PF remarkably inhibited PDGF-BB-induced human PASMCs migration in a dose-dependent manner (n=3). (G) and (H) Flow cytometry analysis showed that PASMCs in the PDGF-BB+PF (1 μM and 10μM) groups exhibited increased apoptosis rates compared to that in the PDGF-BB group. (n=4). Data were presented as the mean ± SEM: (∗) P < 0.05 vs the control group; (#) P < 0.05 vs the PDGF group.

*In vivo* studies showed that PF alleviated MCT-induced pulmonary arterial remodeling by inhibiting PASMC proliferation. To determine whether there is a direct inhibitory effect of PF on PASMC proliferation, the effects of PF on PASMC viability were determined by CCK8 assay. As shown in Figure 6A, PF alone ranged from 0.01 μM to 100 μM had no effects on basal cell viability of PASMCs. However, 0.1 μM to 100 μM PF significantly attenuated PDGF-BB (20 ng/ml)-challenged PASMC viability elevation (Figure 6B). Next, EdU incorporation assay was performed to evaluate the effects of PF on PDGF-BB-induced proliferation of PASMCs. As indicated by the number of EdU^+^ cells in Figure 6C, PF concentration-dependently inhibited PDGF-BB-induced PASMCs proliferation (Figure 6D).

Transwell assay and scratch-wound assay were applied to determine the effects of PF on PDGF-BB-induced PASMC migration. Consistently, either Transwell assay (Figures 6C and 6E) or scratch-wound assay (Figures 6C and 6F) confirmed that PF concentration-dependently suppressed PDGF-BB challenge induced PASMC migration.

Flow cytometry analysis was performed to assess the effects of PF on PDGF-BB-induced PASMC apoptosis. As shown in Figure 6G-H, compared with the control group, there was no significant change in apoptosis in the PDGF-BB group. However, the PASMCs in the PDGF-BB+PF groups showed enhanced cell apoptosis compared to the PDGF-BB group.

### Effect of PF on TGFβ1, IL-1β and TNF-α co-stimulation-induced EndMT in HPAECs

**Figure 7:**
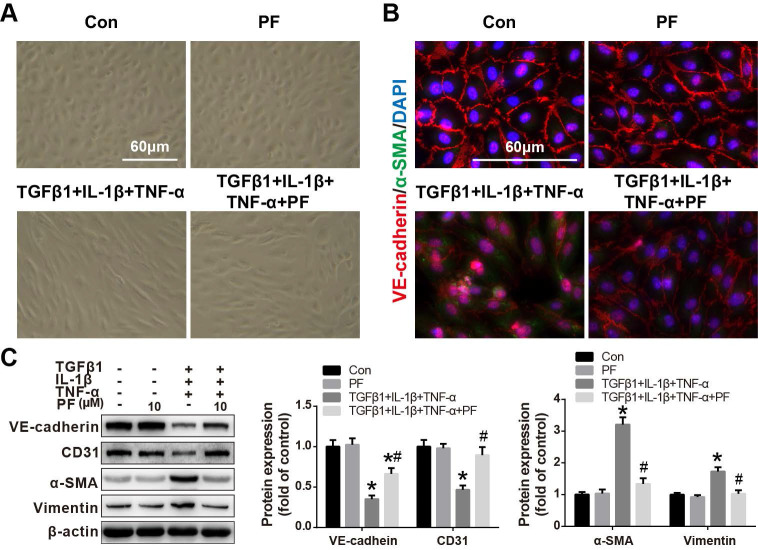
Effect of PF on TGFβ1, IL-1β and TNF-α co-induced EndMT in HPAECs. (A) Morphological examination showed that PF (10 μM) inhibited the spindle-like morphology changes of HPAECs induced by TGFβ1 (5 ng/ml), IL-1β (0.1 ng/ml) and TNF-α (5 ng/ml) (n=5). (B) Double immunofluorescence staining of VE-cadherin and α-SMA shows that PF partially reversed EndMT in the HPAECs after co-cultured with TGFβ1, IL-1β and TNF-α (n=3). (C) Western blotting analysis showed that PF partially reversed the expression of VE-cadherin (n=3), and reversed the expression of CD31 (n=4), α-SMA (n=4), and Vimentin (n=3) co-induced by TGFβ1, IL-1β and TNF-α in HPAECs. Data were presented as the mean ± SEM: (∗) P < 0.05 vs the control group; (#) P < 0.05 vs the TGFβ1 + IL-1β + TNF-α group.

Our *in vivo* results also suggested that inhibiting MCT-induced EndMT contributed to the beneficial effects of PF. Therefore, the effects of PF on TGFβ1, IL-1β and TNF-α-challenged EndMT in HPAECs were further investigated *in vitro*. As shown in Figure 7A, after co-stimulated with TGFβ1 (5 ng/mL), IL-1β (0.1 ng/mL) and TNF-α (5 ng/mL) for 48 h, HPAECs lost their cobble-stone morphology and displayed an elongated and spindle-shaped phenotype, indicating a development of EndMT. However, this morphological change was inhibited by 10 μΜ PF. Immunofluorescence staining showed that TGFβ1, IL-1β and TNF-α treatment resulted in the loss of endothelial marker VE-cadherin on cellular membrane and over-expression of mesenchymal marker α-SMA in cytoplasm, which was alleviated by PF (Figure 7B). Further western blotting analysis confirmed that TGFβ1, IL-1β and TNF-α-challenged down-regulation of endothelial markers (VE-cadherin and CD31) were restored by the treatment of 10 μΜ PF, while the up-regulation of mesenchymal markers (α-SMA and Vimentin) were inhibited by PF in HPAECs (Figure 7C).

### Effects of PF on phosphorylation of TAK1, ERK1/2, p38 MAPK and p65 NF-κB

**Figure 8:**
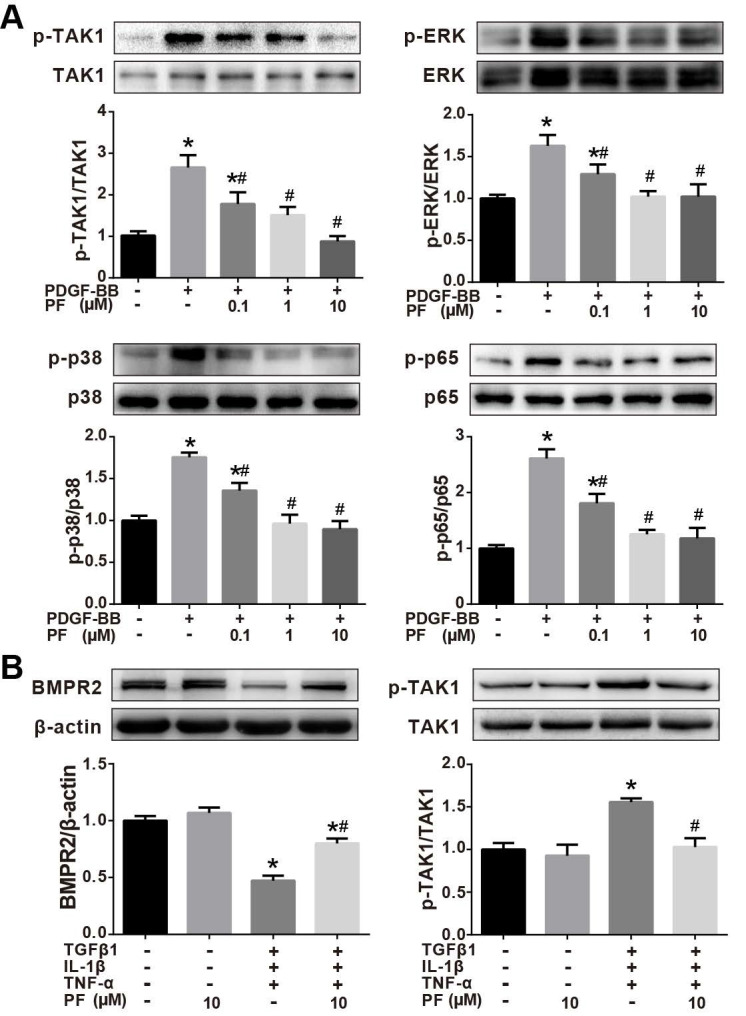
Effects of PF on phosphorylation of TAK1, ERK1/2, p38 MAPK and p65 NF-κB. (A) Western blotting analysis showed that the phosphorylation of TAK1, ERK1/2, p38 MAPK and p65 NF-κB induced by PDGF-BB could be reversed by PF in a dose-dependent manner in PASMCs. (B) Western blotting analysis showed that the phosphorylation of TAK1 induced by TGFβ1, IL-1β and TNF-α could be reversed by PF in HPAECs. PF also partially restored the decreased BMPR2 protein level. Data were presented as the mean ± SEM of 3-4 independent experiments. (∗) P < 0.05 vs the control group; (#) P < 0.05 vs the PDGF or TGFβ1 + IL-1β + TNF-α group.

Our *in vivo* studies showed that PF potently inhibited MCT-induced phosphorylation of TAK1, a key mediator of proinflammatory and stress signals. Therefore, the effects of PF on the phosphorylation of TAK1 and its downstream kinase p38MAPK, ERK1/2, and p65 NF-κB in PDGF-BB-stimulated human PASMCs *in vitro*. As shown in Figure 8A, PDGF-BB triggered a significant phosphorylation of TAK1, ERK1/2, p38 MAPK and p65 NF-κB in human PASMCs, which was inhibited by PF (0.1, 1 and 10 μM) in a dose-dependent way.

The effect of PF on TAK1 phosphorylation was further investigated in TGFβ1, IL-1β and TNF-α-challenged HPAECs. As indicated in Figure 8B, 10 μM PF suppressed down-regulation of BMPR2 and completely reversed phosphorylation of TAK1 in HPAECs induced by TGFβ1, IL-1β and TNF-α challenge.

## Discussion

Pulmonary arterial hypertension is a progressive syndrome caused by many diseases and characterized by increased pulmonary vascular resistance that leading to right ventricular failure and death ultimately. Dysfunction of HPAECs, hyper-proliferation and resistance to apoptosis of PASMCs contribute to the pathophysiology of PAH. Current therapies consist of pulmonary vasodilators that relieve the vasoconstrictive component of PAH while patients still face a poor prognosis due to the ongoing remodeling of pulmonary vasculature. Although new emerging therapies offer an improvement of disease symptoms, their clinical translational application is restricted by systemic toxicity and side effects 14. Thus, there remains an urgent need for novel, effective and safe therapeutics targeting vascular remodeling processes beyond vasodilation. In this study, either preventive or therapeutic administration of PF could alleviate MCT-induced PAH, pulmonary vascular remodeling, right ventricle hypertrophy, and pulmonary vascular inflammation. Recently, we reported that PF treatment prevent vascular remodeling in chronic hypoxia/SU5416-induced PAH rats 9. Together, these data suggest that PF may serve as a promising therapeutic agent for PAH treatment.

In this work, we confirmed the prophylactic and therapeutic effects of PF in MCT-induced PAH. Overall, data from both *in vivo* and *in vitro* studies suggested that the beneficial effects of PF on PAH and pulmonary vascular remodeling can be generally summarized into three aspects: alleviating perivascular accumulation of inflammatory cells and inflammation in lung; inhibiting EndMT in intima; and suppressing over-proliferation of PASMCs in media. Mounting evidence showed that PF presented anti-inflammation and immunomodulatory effects not only in diverse autoimmune diseases (including rheumatoid arthritis, sjogren's syndrome, and systemic lupus erythematosus), but also in different disease models, such as ischemia-reperfusion injury, heart failure, asthma, and pulmonary fibrosis 15. Mechanistically, PF has been reported to inhibit macrophage activation, suppress type I macrophages (M1, pro-inflammatory) activity and enhance type II macrophages (M2, anti-inflammatory) activity, regulate Th1/Th2 balance 16-18. In addition, PF has been documented to inhibit production of pro-inflamamtory mediators (including TNF-α, IL-1β, IL-6, IFN-γ etc.) via suppressing NF-κB activation 19, 20. In past two decades, perivascular accumulation of inflammatory cells including macrophages, T and B lymphocytes, and mast cells are found to be infiltrated around remodeling pulmonary arteries, in both animal models and human PAH. Moreover, inflammatory cytokines, especially IL-1β, IL-6, and TNF-α, play an important role in the development of pulmonary hypertension by increasing vascular reactivity, inducing right ventricular hypertrophy, and enhancing muscularization of the distal arteriolar vessels 21. In our current study, PF treatment significantly inhibited MCT-induced perivascular accumulation of macrophages/monocytes and mast cells, as well as suppressed MCT-induced over-expression of IL-1β, IL-6, and TNF-α in lung. These results indicated that anti-inflammatory effects of PF effectively ameliorate pulmonary vascular remodeling and improved functional outcome in MCT-induced PAH.

In nowadays, it is widely accepted that EndMT is one of the key mechanisms leading to endothelial dysfunction in PAH 10, 22, 23. Generally, EndMT is characterized by the acquisition of mesenchymal cell markers (such as α-SMA, vimentin, and N-cadherin) while loss of endothelial cell markers (such as CD31, VE-cadherin, and eNOS) in endothelium. EndMT can be induced by various intrinsic or extrinsic factors including cytokines, inflammation, hypoxia, and radiation 24. Recently, it has been documented that BMPR2 signaling dysfunction, TGFβ1 stimulation, as well as inflammatory challenge are key risk factors to EndMT in different experimental PH models 25-27. In our study, we found that MCT administration led to an up-regulation of TGFβ1 but a downregulation of BMPR2 in lung. Moreover, MCT also led to a significant inflammation in lung as indicated by massive CD68^+^ macrophages and mast cells infiltration and up-regulation of inflammatory factors IL-6, IL-1β, and TNF-α. However, the aforementioned pathology induced by MCT could be blocked by preventive treatment of PF, thus alleviating EndMT in lung. This result was further confirmed by the *in vitro* study that PF effectively inhibited TGFβ1, IL-1β and TNF-α co-induced EndMT of HPAECs by restoring BMPR2 expression and inhibiting downstream TAK1 phosphorylation. It should be noted that high levels of TGFβ1 could induced a significant decrement of BMPR2 in human pulmonary microvascular blood vessel endothelial cells, while BMPR2 reduction sufficiently promotes EndMT in pulmonary arterial hypertension 28. Therefore, these data from either *in vivo* or *in vitro* studies suggested that PF could inhibit TGFβ1-TAK1 signaling and inflammation, which subsequently maintained BMPR2 function and suppressed EndMT in lungs of MCT-injected rats.

Hypertrophy, proliferation, migration, and resistance to apoptosis of smooth muscle cells contribute to pulmonary arterial media remodeling in PAH. Growth factor signaling, especially the platelet-derived growth factor receptor (PDGFR) signaling, plays a pivotal role in promoting PASMCs proliferation. Generally, PDGF-BB is regarded as the most potent mitogen for PASMCs proliferation and migration. Increased expression of PDGF and PDGFRs was found in the lungs of patients and animal models with PAH 29. Pharmacological inhibition of the PDGFR signaling could reverse the progression of PAH by preventing PASMCs proliferation-mediated pulmonary vascular remodeling in animal models 30. There are evidence showing that PF inhibits smooth muscle cell proliferation and migration induced by PDGF‑BB in human airway smooth muscle cells and vascular smooth muscle cells 31, 32. Our *in vitro* study confirmed that PF effectively suppressed PDGF-BB induced PASMCs proliferation, migration and apoptosis resistance in a concentration-dependent manner. It is likely that inhibition of pulmonary arterial smooth muscle proliferation and vascular remodeling is predominantly a direct effect of PF, because PF effectively inhibited proliferation and apoptosis resistance of PASMCs both* in vivo* and *in vitro*.

In the past decade, TAK1 has been clearly demonstrated as a key player in a wide range of physiological processes including inflammation, immune system homeostasis and neural development 33-35. Increased expression and phosphorylation of TAK1 has been reported in MCT induced PAH rat models 36. In PASMCs, it was reported that BMPR2 dysfunction promotes the activation of MAPK pathways via TAK1, leading to a pro-proliferative and anti-apoptotic response 37. Inhibition of the TAK1-MAPK axis rescues abnormal proliferation and apoptosis in these cells 37. In our study, we found that PF decreased the constitutive activation of TAK1 in lungs from MCT-treated rats. Our *in vitro* studies further showed that PF successfully inhibited phosphorylation of TAK1 not only in PDGF-BB-challenged PASMCs but also in TGFβ1 and IL-1β-stimulated endothelial cells. Thus, our data from both *in vivo* and *in vitro* studies suggested that PF could suppress the development of PAH by sustained inhibition of TAK1.

It is well established that MAPKs are the most important kinases involved in the proliferation, migration, and inflammation of PASMC induced by PDGF-BB 38. In PAH, the ERK1/2-MAPK mediates mitogen-induced proliferation of PASMCs, while p38-MAPK involves the transcription of a range of pro-inflammatory genes, including TNF-α, IL-1β and IL-6 39, 40. In airway smooth muscle cells, PDGF could induce a significant proliferation by activating ERK1/2, which was abolished by pharmacological inhibition of TAK1, suggesting TAK1-MAPK is a downstream signaling of PDGF. In current studies, PF attenuated PDGF-BB-induced phosphorylation of TAK1, as well as p38 and ERK1/2 in PASMCs, indicating inhibition of TAK1-p38/ERK1/2-MAPK signaling may contribute to the therapeutic effects of PF.

NF-κB is a key transcription factor responding to either PDGF or TGFβ 41. It has been well documented that NF-κB pathway is involved in multiple processes of the development and progression of PAH, such as inflammation, EndMT, and vascular remodeling 42. For example, pharmacological inhibition of NF-κB suppressed transcriptions of plasminogen activator inhibitor 1 and monocyte chemotactic protein-1 in MCT-treated rat, and thereafter attenuated inflammation and pulmonary vascular remodeling in lung 43. Moreover, NF-κB is a well-documented target of TAK1 in inflammatory cytokine signaling such as IL-1β, TNF-α, or TGFβ 44. In cultured PASMCs, we found that PF suppressed PDGF-induced phosphorylation of either TAK1 or p65 NF-κB. Although several studies have shown that PF also affected other signal pathways (such as Akt), these results suggested that TAK1-NF-κB is another important signaling pathway involved in the therapeutic mechanism of PF.

## Conclusion

In summary, our research suggested that PF is a potential therapeutic drug for PAH. Either preventive or therapeutic administration of PF could alleviate MCT-induced PAH by attenuating vascular remodeling, alleviating inflammation and EndMT in lung. The underlying mechanism of PF is associated with the inhibition of TAK1-MAPK/NF-κB pathways, which may be promising targets for the therapeutic intervention of PAH.

## Figures and Tables

**Table 1:** The primer sequences of targeted RNA.

Gene primer	Species		Sequence (5' to 3')
*IL-6*	Rat	Forward	AGACTTCACAGAGGATACCACCCAC
		Reverse	CAATCAGAATTGCCATTGCACAA
*IL-1β*	Rat	Forward	ACAAGGAGAGACAAGCAACGACAA
		Reverse	ACAAGGAGAGACAAGCAACGACAA
*TNF-α*	Rat	Forward	GCGTGTTCATCCGTTCTCTACC
		Reverse	GCGTGTTCATCCGTTCTCTACC
*β-actin*	Rat	Forward	CTGAACCCTAAGGCCAACCG
		Reverse	GACCAGAGGCATACAGGGACAA
